# Oxidative alterations in exfoliated oral mucosa cells of patients with major depressive disorder

**DOI:** 10.1016/j.jobcr.2025.01.026

**Published:** 2025-02-10

**Authors:** Lukas Mendes de Abreu, Cintia Rodrigues da Silva, Ana Laura Ferreira Bortoleto, Giovana Barros Nunes, Matheus Martins Gracia, Rafael Akira Tzanno Murayama, Daniel Galera Bernabé, Gisele Zoccal Mingoti

**Affiliations:** a Oral Oncology Center, São Paulo State University (Unesp), School of Dentistry, Campus Araçatuba, São Paulo, Brazil; b São Paulo State University (UNESP), Graduate Program in Veterinary Medicine, School of Agrarian and Veterinary Sciences, Campus Jaboticabal, São Paulo, Brazil; c Specialized Psychosocial Care Center (CEAPS), Araçatuba, São Paulo, Brazil; d Department of Diagnosis and Surgery, São Paulo State University (Unesp), School of Dentistry, 1193 José Bonifácio St Araçatuba, SP 16050-015, São Paulo, Brazil; e São Paulo State University (UNESP), School of Veterinary Medicine, Laboratory of Reproductive Physiology, Campus Araçatuba, São Paulo, Brazil

**Keywords:** Major depressive disorder, Depression, Oxidative stress, Oral mucosa, Oral disease

## Abstract

**Objectives:**

This study aimed to investigate oxidative stress markers in the oral mucosal cells of individuals diagnosed with major depressive disorder (MDD).

**Methods:**

A case-control design was used, including twenty patients diagnosed with MDD, based on the Diagnostic and Statistical Manual of Mental Disorders (DSM-5) criteria, and twenty healthy controls. Oral exfoliated cells were collected from all participants. Intracellular levels of reactive oxygen species (ROS), mitochondrial membrane potential (ΔΨm), caspase-3 and -7 activity, and reduced glutathione (GSH) were measured in Arbitrary Fluorescence Units (AFU).

**Results:**

The MDD group demonstrated significantly elevated intracellular ROS levels (p = 0.0012) and caspase-3 and -7 activity (p = 0.0171) in comparison to the control group. Additionally, a decrease in ΔΨm expression was observed in the oral cells of MDD patients (p = 0.0265), whereas GSH expression levels did not differ significantly between the two groups (p = 0.8908).

**Conclusions:**

The findings indicate heightened oxidative stress in the oral exfoliated cells of individuals with MDD. This study supports the potential use of oral cells as a non-invasive biomarker source for assessing oxidative stress in depressive disorders.

## Graphical abstract

**Figure:**
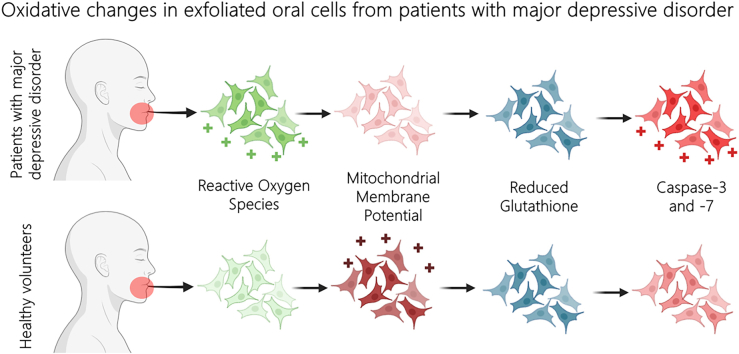

## 1 Introduction

Major Depressive Disorder (MDD) is a chronic and recurrent mood disorder commonly found throughout the world.^1^^,^^2^ This disorder affects at least 3.8 % of the population and is twice as common in females as in males.^3^^,^^4^ In addition to being characterized by the occurrence of persistent emotional symptoms, MDD can affect cognitive functions and compromise physical health.^1^^,^^4^ The pathogenesis of MDD involves genetic, environmental, psychological, and biological factors,^3^ including molecular mechanisms that lead to disruptions in the metabolism of neurotransmitters such as norepinephrine, serotonin, and dopamine.^5^^,^^6^ Its occurrence and progression are associated with elevated systemic levels of pro-inflammatory cytokines and mitochondrial dysfunction,^6^, ^7^, ^8^ contributing to the oxidative stress observed in patients with MDD.^7^, ^8^, ^9^

Oxidative stress refers to an imbalance between the oxidant and antioxidant systems in favor of the oxidants.^10^ This imbalance can result in the degradation of essential biological structures for organic cell functioning, including oxidative modifications of lipids, proteins, DNA, enzymes, and the activity of certain genes.^10^ Patients diagnosed with MDD have elevated systemic levels of enzymes involved in the production of reactive oxygen species (ROS) and reactive nitrogen species (RNS) compared to healthy controls,^8^^,^^9^^,^^11^ leading to an increase in the levels of xanthine oxidase and ROS in the blood and brain of MDD patients. Moreover, studies have demonstrated elevated levels of oxidative damage products in various biological samples such as peripheral blood, red blood cells, mononuclear cells, urine, cerebrospinal fluid, and postmortem brains of patients with MDD.^8^, ^9^, ^10^

Oxidant and antioxidant systems play crucial roles in the physiological functions of oral mucosal cells.^12^ Oxidative stress has been implicated in the pathogenesis or exacerbation of several oral diseases, including periodontal disease, recurrent aphthous ulcers, salivary gland diseases, autoimmune disorders, oral leukoplakia, and squamous cell carcinoma.^12^, ^13^, ^14^ This involvement is related to the constant exposure of the oral mucosa to endogenous and exogenous oxidative stress sources.^12^^,^^13^^,^^15^^,^^16^ Furthermore, saliva spreads dissolved antioxidants and oxidants from different sources, reaching oral cells.^12^^,^^16^ However, there is a lack of knowledge regarding the occurrence of oxidative stress-related changes in the oral mucosa of patients diagnosed with MDD.

In this way, knowing that there is an increased oxidative stress condition in individuals with MDD^8^^,^^9^ and that the imbalance of the oxidant-antioxidant state damages the oral mucosa cells,^15^ it becomes essential to investigate the occurrence of redox disorders in the oral mucosa in a MDD condition. Therefore, to test the hypothesis that patients with MDD exhibit increased oxidative stress in oral mucosal cells, this study aimed to evaluate the expression of molecules associated with oxidative stress in exfoliated oral cells from patients with MDD compared to those from healthy individuals. To our knowledge, this is the first study designed to investigate specific molecular changes related to oxidative stress in oral exfoliated cells of patients with MDD.

## 2 Materials and methods

### 2.1 Ethics considerations

This study was approved by the Committee of Human Studies of São Paulo State University (UNESP), School of Dentistry, Araçatuba, São Paulo, Brazil (protocol number 5.719.154), and was conducted according to the principles of the Declaration of Helsinki on clinical studies with humans.

### 2.2 Study design

A case-control observational study was carried out to assess oxidative stress in exfoliated oral cells of patients diagnosed and undergoing treatment for MDD in a single institution specializing in the treatment of psychiatric disorders. The study included patients with MDD (MDD group) and matched healthy volunteers (control group). The method used to evaluate the expression of oxidative stress in exfoliated oral cells was previously detailed and validated by our research group.^14^ A sample size calculation was carried out using G∗Power software (Version 3.1.9.7; Heinrich-Heine-Universität Düsseldorf, Düsseldorf, Germany) considering the difference between means for independent groups (*t*-test) with an alpha error of 5 % and test power of 80 %.^17^ The calculation indicated a minimum of 17 participants per group. Considering potential data loss, 20 participants were recruited for each group.

### 2.3 Patients and volunteers

A comprehensive evaluation was conducted using the medical records of patients undergoing treatment for MDD at the Specialized Psychosocial Care Center (CEAPS) in Araçatuba, São Paulo, Brazil. The inclusion criteria for the MDD group required participants to be male or female, over 18 years of age, and diagnosed with MDD by a single psychiatrist, following the criteria established in the fifth edition of the Diagnostic and Statistical Manual of Mental Disorders (DSM-5). The diagnosis was based on the presence of a depressed mood and/or loss of interest for at least two weeks, accompanied by symptoms such as changes in sleep, appetite, and energy levels. Depression severity was assessed using the Hamilton Rating Scale for Depression (HAM-D), and only patients with mild (8–13) to moderate (14–18) scores were included.^18^ All participants in the MDD group were undergoing pharmacological treatment with selective serotonin reuptake inhibitors (SSRIs), with individualized dosages adjusted according to their psychiatric evaluations. Although the potential impact of SSRIs on oxidative stress markers was not specifically addressed in this study, the inclusion of patients exclusively using SSRIs aimed to control variability arising from different pharmacological treatments. This approach reduced potential confounding factors associated with varying mechanisms of action of different drug classes. Furthermore, the stable treatment regimens of all participants minimized acute pharmacological effects that could influence oxidative imbalance.

For the control group, volunteers were recruited and matched to the MDD group by sex, age, and general health status. Both groups underwent a rigorous exclusion process. All participants completed a structured questionnaire to collect sociodemographic data, followed by a thorough intraoral examination to assess oral health and ensure the absence of lesions or mucosal diseases. Exclusion criteria included smoking, excessive alcohol consumption, illicit drug use, poor oral hygiene, active oral diseases, or cognitive impairments that could interfere with clinical or psychiatric assessments. Additionally, any medical condition that could compromise general health or the study's outcomes was excluded, ensuring that only healthy individuals were included. After applying the inclusion and exclusion criteria, a total of 40 participants (20 in each group) were included in the study. The MDD group consisted of 16 women (80 %) and 4 men (20 %), with an average age of 51 years, and the control group was age- and sex-matched.

### 2.4 Evaluation of oxidative stress in oral exfoliated cells

#### 2.4.1 Collection of exfoliated oral cells

Cell collection from participants in the MMD and control groups was carried out in the morning, between 09:00 a.m. and 11:00 a.m., with one participant from each group always being collected on the same day. Participants were instructed not to eat or brush their teeth for at least 2 h before sample collection. Immediately before cell collection, they were instructed to rinse their mouths three times with distilled water. The cells were collected using a cytobrush (Adlin, Santa Catarina, BR), from the lateral border of the tongue and floor of the mouth, which are the regions most affected by oral cancer.^15^ The cytobrush was applied to the surface of the oral mucosa with a circular expansion movement starting at the lateral border of the tongue and gradually increasing the circumference until reaching the floor of the mouth. To maximize cell sampling and eliminate any bias that could be caused by sampling from only one side, the cells were exfoliated in the regions of interest on the right and left sides using different brushes. Analyzes were carried out immediately after cell collection.

#### 2.4.2 Cell preparation

After exfoliation, the brushes were placed in a single 15 mL conical tube (Corning, Condado de Steuben, NY, EUA) containing 5 mL of fresh phosphate buffered saline (PBS) with 1 mg/mL of polyvinyl alcohol (PBS/PVA; Sigma-Aldrich, St. Louis, MO, USA). The cytobrushes were then vigorously rotated to dislodge and release the cell to the bottom of the tube. The tube containing cells from both sides of the oral mucosa was centrifuged at 1160 *g* for 5 min at 37 °C. The supernatant was discarded and replaced with 5 mL fresh PBS/PVA. The washing procedure was repeated twice. Next, cells were counted using a hemocytometer and diluted in PBS/PVA to a final concentration of 50,000 cells/mL. After setting the cell concentration, 2 mL of cell suspension was retrieved, and the volume was divided equally into two microtubes (1 mL of cell suspension in each microtube). Microtubes were centrifuged at 1160 *g* for 5 min at 37 °C. At the end of the centrifugation, the supernatant was removed and the pellet containing the cells was subjected to staining protocols.

#### 2.4.3 Simultaneous evaluation of intracellular expression of reactive oxygen species and mitochondrial membrane potential

The intracellular content of ROS (H_2_O_2_, HO•, and ROO•) and mitochondrial membrane potential (ΔΨm) were concurrently assessed using the probes H_2_DCFDA (6-carboxy-2′,7′-dichlorodihydrofluorescein diacetate; Molecular Probes, OR, USA) and MitoTracker Red (CMXRos, Molecular Probes), respectively, using the same protocol previously described by Abreu et al.^14^ After staining, the cells were evaluated immediately under a microscope equipped with epifluorescence (IX51, Olympus; 20 × magnification) running the Q-Capture Pro Image Software (Media Cybernetics, Inc., Version 5.0.1.26). The excitation and emission wavelengths were, respectively: 615 nm and 587 nm for Image-iT LIVE Red Caspase-3 and -7, and 404 nm and 526 nm for ThiolTracker Violet Data were expressed in arbitrary fluorescence units (AFU).

#### 2.4.4 Simultaneous evaluation of intracellular glutathione expression and caspase-3 and -7

The intracellular levels of reduced glutathione (GSH) and caspase-3 and -7 were concurrently measured using the fluorescent probes ThiolTracker Violet (Glutathione Detection Reagent; Molecular Probes) and Image-iT LIVE Red Caspase-3 and -7 (Molecular Probes), respectively. The intracellular expression of GSH and caspase-3 and -7 was determined using the method previously described by de Abreu et al.^14^ with some modifications. In summary, a fresh solution was prepared with 20 μM ThiolTracker Violet and 30x fluorescent inhibitors of caspase-3 and -7, diluted in 150 μL of fresh PBS/PVA. Unstained oral cells underwent incubation with this solution for 45 min at 37 °C in the dark, and then washed twice with fresh PBS/PVA. The cells were evaluated immediately under a microscope with epifluorescence at excitation and emission wavelengths of, respectively, 615 nm and 587 nm for Image-iT LIVE Red Caspase-3 and -7, and 404 nm and 526 nm for ThiolTracker Violet. Data were expressed in AFU.

### 2.5 Statistical analysis

Data were analyzed by paired *t*-test using GraphPad Prism 8.21 (GraphPad Software Inc., San Diego, CA, USA). The chi-squared test was used to explore associations between demographic and medical variables between groups. For each biomarker, AFUs were composed of an average of 100 oral cells from each participant. Normality testing was determined using the D'Agostino-Pearson test, and the paired *t*-test was employed to assess differences in AFUs for each biomarker between the groups. Differences were considered statistically significant when p < 0.05.

## 3 Results

### 3.1 Intracellular expression of reactive oxygen species

**Fig. 1:**
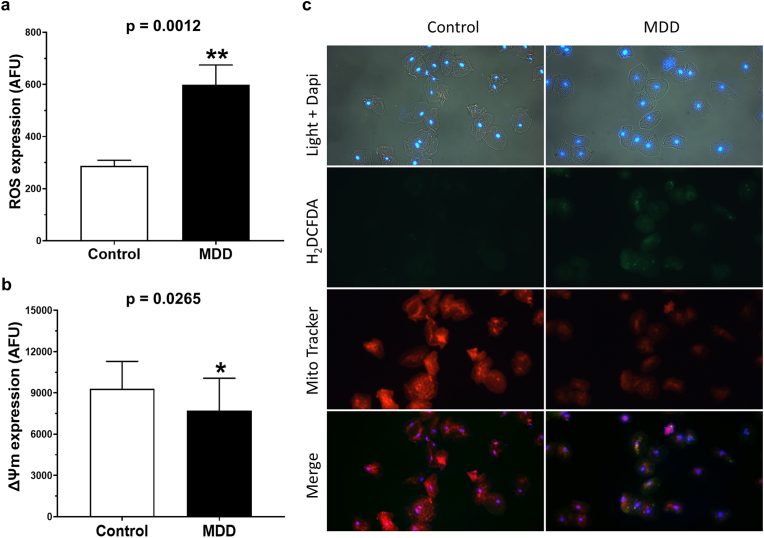
Intracellular expression of reactive oxygen species (ROS) and mitochondrial membrane potential (ΔΨm) in exfoliated oral cells collected from patients diagnosed with major depressive disorder (MDD) and control participants without psychiatric disorders. (a) Graph showing the mean ± standard error of the mean (SEM) for fluorescence intensity (pixels) of ROS in cells stained with H_2_DCFDA, expressed in arbitrary fluorescence units (AFU). MDD group: n = 2000 cells; control group: n = 2000 cells. ∗∗p < 0.001 indicates statistically significant difference. (b) Graph showing the mean ± SEM for fluorescence intensity (pixels) of ΔΨm in cells stained with MitoTracker Red®, expressed in AFU. MDD group: n = 2000 cells; control group: n = 2000 cells. ∗p < 0.05 indicates statistically significant difference. (c) Representative images of exfoliated oral cells under light microscopy (gray) and fluorescence microscopy with DAPI (blue-stained nuclei), H_2_DCFDA (green fluorescence indicating ROS), and MitoTracker Red (red fluorescence indicating ΔΨm). (For interpretation of the references to colour in this figure legend, the reader is referred to the Web version of this article.)

The intracellular expression of ROS was quantified in exfoliated oral cells and it was observed higher expression (p = 0.0012) of intracellular ROS in exfoliated oral cells from patients with MDD compared to the control group (MDD: 599.3 ± 75.8 AFU vs control: 288.0 ± 21.1 AFU; Fig. 1A). Representative photomicrographs of exfoliated oral cells stained for ROS evaluation are shown in Fig. 1C.

### 3.2 Intracellular expression of mitochondrial membrane potential

The results demonstrated that exfoliated oral cells from patients with MDD exhibited lower expression of ΔΨm than those from the control group (MDD: 7703.3 ± 527.3 AFU vs control: 9299.6 ± 443.6 AFU; p = 0.0265). Data are summarized in Fig. 1B, while representative photomicrographs of stained exfoliated oral cells for evaluation of ΔΨm are shown in Fig. 1C.

### 3.3 Intracellular expression of reduced glutathione

**Fig. 2:**
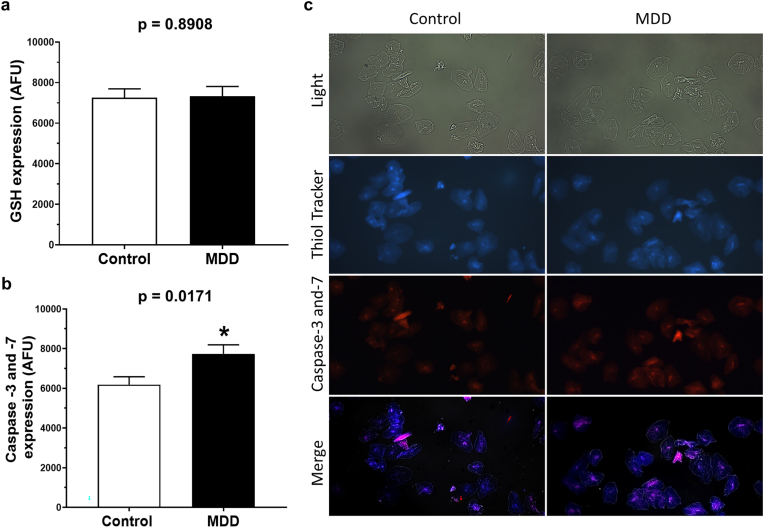
Intracellular levels of reduced glutathione (GSH) and caspase-3 and -7 activity in exfoliated oral cells from patients with major depressive disorder (MDD) and control participants without psychiatric disorders. (a) Graph showing the mean intensities ± SEM for fluorescence signals (pixels) of GSH, measured with ThiolTracker™ Violet probe, expressed in AFU. MDD group: n = 2000 cells; control group: n = 2000 cells. ∗p < 0.05 indicates statistically significant difference. (b) Graph showing the mean intensities ± SEM for fluorescence signals (pixels) for caspase-3 and -7 activity, measured with Image-iT LIVE Red Caspase-3 and -7 probe, expressed in AFU. MDD group: n = 2000 cells; control group: n = 2000 cells. ∗p < 0.05 indicates statistically significant difference. (c) Representative images of exfoliated oral cells under light microscopy (gray) and fluorescence microscopy with ThiolTracker™ Violet (blue, indicating GSH) and Image-iT LIVE Red Caspase-3 and -7 (red, indicating apoptosis). (For interpretation of the references to colour in this figure legend, the reader is referred to the Web version of this article.)

The intracellular expression of GSH in oral exfoliated cells was similar between the MDD and control groups (MDD: 7336.3 ± 472.9 AFU vs control: 7258.7 ± 434.7 AFU; p = 0.8908), as demonstrated in Fig. 2A. Representative photomicrographs of stained exfoliated oral cells for evaluation of GSH levels are shown in Fig. 2C.

### 3.4 Intracellular expression of caspase-3 and -7 activity

The intracellular expression of caspase-3 and -7 (Fig. 2B) was higher in exfoliated oral cells of patients with MDD than in control volunteers (MDD: 7735.4 ± 452.8 AFU vs control: 6183.1 ± 398.1 AFU; p = 0.0171). Representative photomicrographs of stained exfoliated oral cells for evaluation of intracellular levels of caspase-3 and -7 activity are shown in Fig. 2C.

## 4 Discussion

It is well known that patients diagnosed with MDD have elevated levels of systemic oxidative stress.^5^ While increases in oxidative stress levels have been reported in fluids like serum/plasma and urine from MDD patients,^9^^,^^19^ these changes had not yet been demonstrated in oral mucosal epithelial cells. Although a minimal level of ROS is indispensable for cell survival, high levels of ROS in cellular compartments result in slow cell growth, cell cycle arrest and apoptosis.^10^ In the present study, we demonstrated that exfoliated oral cells from patients with MDD exhibit increased expression of ROS compared to volunteers without psychiatric disorders.^20^ The molecular events underlying oxidative stress in MDD patients remain elusive. However, numerous studies indicate elevated levels of pro-inflammatory cytokines in their blood, contributing to the genesis and buildup of ROS.^21^^,^^22^

Excessive ROS generation and accumulation have the potential to harm various cellular components, such as cell membranes and mitochondria, culminating in mitochondrial dysfunction.^23^^,^^24^ Damaged mitochondria exhibit a loss of Δψm, triggering the opening of mitochondrial permeability transition pores and the release of cytochrome *c* into the cytoplasm.^20^^,^^24^ As cytochrome *c* is an important mediator of apoptosis, its release into the cytosol can trigger programmed cell death.^20^^,^^24^ Thus, the reduction in Δψm has been reported to be an early event and a possible cause of programmed cell death.^25^ Our results suggest that the intracellular increase in ROS levels in the oral exfoliated cells of patients with MDD led to mitochondrial dysfunction by loss of Δψm in these patients’ cells. Although studies in MDD patients have not yet investigated the ΔΨm, it is consistent among the different studies that MDD is triggered, at least in part, by mitochondrial dysfunction.^20^^,^^23^^,^^24^^,^^26^, ^27^, ^28^ These studies suggest that increased ROS may contribute to mitochondrial dysfunction and neuronal death in patients with MDD.^20^^,^^23^^,^^24^^,^^26^, ^27^, ^28^, ^29^, ^30^

Caspases-3 and -7 are the main effector caspases involved in the intrinsic pathway of apoptosis, which is triggered by cellular stress, damage, or programmed cell death.^31^ Our findings indicate an elevated intracellular expression of caspases-3 and -7 in oral epithelial cells from MDD patients compared to control volunteers. Similarly, increased caspase-3 and -7 activity was observed in the hippocampus of rats subjected to the depression model.^32^, ^33^, ^34^ While caspase activity has not been directly reported in MDD patients, a previous study revealed an association between the upregulation of 20 genes linked to the apoptosis pathway in MDD patients.^35^ The heightened caspase activity observed in oral cells from MDD patients in our study may be linked to the ΔΨm loss, potentially leading to the release of intermembrane cytochrome *c* into the cytoplasm.^36^^,^^37^ According to the literature, this death pathway involves the binding of cytochrome *c* to the apoptotic protease activating factor 1, recruiting caspase-9 and forming apoptosomes, ultimately activating executor caspases-3 and -7.^31^ This cascade results in protein cleavage, DNA fragmentation, and cell death.^20^^,^^31^

Redox imbalance and the consequent progression to cell apoptosis can be prevented by antioxidant actions,^38^ and GSH is one of the most important intracellular antioxidants in the cell.^10^ The relationship between MDD and antioxidant levels in the body is complex and not yet fully understood.^5^^,^^9^^,^^19^^,^^39^ Some studies suggest that depression may be associated with lower antioxidant levels, while others have not shown any significant association.^9^^,^^19^^,^^39^^,^^40^ In our study, the intracellular expression of GSH in exfoliated oral cells of patients with MDD was similar to that of voluntary controls. It is important to understand the reasons for these differing findings, as patients with reduced GSH levels in other studies often presented with severe MDD.^41^^,^^42^ Interestingly, some studies, similar to ours, did not find significant differences in GSH levels between healthy controls and depressed patients, whether treated or untreated with SSRIs.^43^^,^^44^ This suggests that SSRI treatment may not be a significant confounding factor. It is more likely that patient heterogeneity plays an important role, and in this context, it is worth noting that our patients were generally not severely depressed nor treatment-resistant. Furthermore, another explanation for our results may be due to saliva, which is an important source of antioxidants in the oral cavity and may contribute to the local maintenance of antioxidant levels in exfoliated oral cells.^16^^,^^45^ Therefore, based on our results, it can be suggested that GSH expression was not high enough to decrease ROS expression in MDD patients, leading to the loss of ΔΨm expression and increased caspase-3 and -7 in their exfoliated oral cells.

Our findings should be interpreted within the context of several limitations. The study was confined to exfoliated oral cells, potentially limiting the comprehensive assessment of oxidative stress compared to other biological matrices, such as serum or plasma, which have received more extensive research focus. Future investigations should encompass a broader array of biological matrices to enhance understanding. Furthermore, the long-term clinical implications of heightened intracellular oxidative stress in the oral mucosa of MDD patients remain uncertain and warrant further exploration through both pre-clinical and clinical studies.

## 5 Conclusions

The current study demonstrates that the exfoliated cells of the oral mucosa are practical and non-invasive additional resources to assess oxidative stress in patients with MDD. Our findings showed a significant increase in ROS levels and caspase-3 and -7 activity and a significant decrease in ΔΨm levels in the oral epithelial from the patients with MDD. Taken together, the results reveal for the first time the occurrence of oxidative stress in exfoliated cells of the oral mucosa of patients with MDD. Our data suggest the use of oral cells as a method for assessing oxidative imbalance in patients with MDD.

## Author contributions

All authors contributed to the study conception and design. LMA designed the research, conducted the experiments, analyzed the data, and wrote the manuscript. CRS, ALFB and GBN participated in the experiments. MMG participated in the conception and design of the study, in data acquisition. RATM participated in the conception and design of the study, in the evaluation of patients with MDD and reviewed the manuscript. GZM and DGB designed the research, supervised the experiments, analyzed the data, and reviewed the manuscript. All authors read and approved the final manuscript.

## Ethical approval and consent to participate

Ethics approval and consent to participate in this study was conducted according to the principles of the Declaration of Helsinki on clinical studies with humans. Ethics approval was granted by the Ethics Committee of Human Studies of São Paulo State University (Protocol number 5.719.154). Written informed consent was obtained from all individual participants included in the study.

## Data availability statement

The data that support the findings of this study are available from the authors upon reasonable request.

## Funding information

This work was supported by the 10.13039/501100001807São Paulo Research Foundation (FAPESP, Brazil, Grants No. 2019/11174-6) and the 10.13039/501100002322Coordenação de Aperfeiçoamento de Pessoal de Nível Superior (CAPES, Brazil, Finance Code 001).

## Declaration of competing interest

The authors declare that they have no known competing financial interests or personal relationships that could have appeared to influence the work reported in this paper.
